# 

**Published:** 1891-05-02

**Authors:** 


### NOTICE TO CORRESPONDENTS.

All MS., Letters, Books for Review, and other matters intended for the Editor should be addressed The Editor, The Lodge, Porchester Square, London, W.
The Editor cannot undertake to return rejected M.S., even when accompanied by stamped directed envelope.

### Notice to Advertisers and Subscribers.

All Advertisements, Orders for Copies of The Hospital, and Business Communications should be addressed to The Publisher, *not to the Editor*, The Hospital, 140, Strand, W.C.
The Cost of Subscription, post free, is as follows:—Per week, 1½d.; per quarter, 1s. 8d.; per half-year, 3s. 3d.; per annum, 6s. 6d. Foreign:—Per annum, 8s. 8d.; payable, in all cases, in advance.

**NOTICE.**—The Index for Vol. IX., (ending March, 1891) is on sale at the Office of this Journal, price 2½d., post free.

### Scraps and Gleanings.

The collections in the churches and chapels of Chatham on Hospital Sunday, amounted to nearly £200.

A fancy-dress ball was given in Limerick on the 17th ult., in aid of the funds of Barington's Hospital.

April 18th was Hospital Saturday in Sheffield. The first returns give the total subscriptions as £920 18s. 11d.

The St. Louis Hospital, in Paris, has an income of over one hundred thousand a-year in landed estates throughout France.

The census returns for Glasgow show that the population numbers 564,975. This is an increase over the census of 1881 of 53,560.

The Fishmongers' Company has granted twenty-five guineas to Mrs. Horlock's Holiday Homes for Working Girls at Southend-on-Sea.

The Home for children of the middle class, orphans and others, which was established at Crayford five years ago, has removed to Ramsgate.

Most of the 34 persons, bitten by a rabid wolf, at Rohozna, will proceed to Bucharest, to be treated at the Pasteur Institute in that city.

At the Leeds Dispensary a large number of influenza cases are under treatment, and several patients have also been received at the Infirmary.

An anonymous donor is about to present an oil painting of the late Duke of Albany to the National Hospital for Diseases of the Heart and Paralysis, Soho Square.

The Princess Beatrice will preside at the annual meeting of the Children's Country Holidays Fund, which will be held at the Hotel Metropole, on May 12, at 3.30 p.m.

The annual festival dinner of the Metropolitan Hospital was held on April 22nd. The Chairman, Sir Julian Goldsmid, M.P., announced subscriptions amounting to £2,455.

Scarlet fever is raging in several villages in East Kent, and in spite of the efforts of the sanitary authorities the epidemic is increasing. The outbreak has necessitated the closing of one school, and it is feared that the other schools will have to follow suit.

Miss Elizabeth Orr Bell, who died lately, bequeathed the whole of the residue of her estate, after payment of certain legacies, to "General" Booth for the Salvation Army. The residue, it is believed, will amount to between £60,000 and £70,000.

The Prince of Wales presided at the festival dinner of the Eastern Counties Asylum for Idiots on the 24th ult. The subscriptions amounted to £5,920, including £1,000 each from two "friends," and 100 guineas from the proprietors of the Hotel Métropole.

Before the Leeds and West Riding Medico-Chirurgical Society, at a recent meeting, it was reported that a member had been called to a man in a public-house who was comatose and much collapsed. He had had very little alcohol, but had smoked in two hours two twopenny and six penny cigars. He recovered after an injection of ether.

The annual summary of the Registrar-General for 1890 estimates the population of London in the middle of the year at 4,421,661 persons, and states that the birth-rate was in the proportion of 29·1 annually per 1,000 of the estimated population. This is by far the lowest birth-rate as yet recorded in London, the rate having fallen continuously year by year since 1879, when it was 35·5 per 1,000.

At the meeting of the Court of Common Council on the 21st ult., the Sanitary Committee, on the reference as to the sanitary condition of St. Bartholomew's Hospital, recommended that notice should be served upon the authorities to amend the drainage, &c., as required. A report was read from the surgical medical staff at St. Bartholomew's Hospital stating that a searching inquiry by a number of the medical staff had failed to show that the recent outbreak of diphtheria was in any way due to the defective sanitary arrangements.

### The Lords' Committee.

The Lords' Select Committee resumed their inquiry on April 23rd. Dr. H. Fenwick was recalled, and spoke of the desirability of greater co-operation between general and special hospitals, as he said such co-operation would enable a great extension of the educational power of special hospitals. All hospitals should be licensed by a body of the heads of the profession, who were in a position to judge without bias of the merits and necessity of starting a hospital.—Mr. Arthur Lucas, vice-chairman of the Hospital for Sick Children, Great Ormond Street, which was founded in 1852, said he supposed it would be called a special hospital, but really it was a general hospital for children. All new hospitals should be licensed, and the licensing authority should not be composed of doctors, as they would probably be influenced by professional jealousies. The witness stated that there were ten cots endowed in perpetuity by the payment of £1,000 by some friends of the hospital, and seven cots endowed for life by the payment of £300.—Dr. W. B. Cheadle, senior surgeon of the same hospital, was next examined; and spoke in support of special and separate hospitals for children, which he considered had many advantages. He also gave evidence of the great mortality of children as compared with adults.—Mr. R. Gilbert said that he was the secretary of the West London Hospital at Hammersmith. He received £250 a-year, with house, rates and taxes. It was a general hospital without a school. Every patient was required to bring a letter, but, if an urgent case, an applicant would be treated, even if he had not a letter. He had made many inquiries as to the circumstances of the patients, and he did not think it was abused to any extent.

### THE STUDENT OF MEDICINE.

[All communications for this Supplement should be addressed to The Editor of The Hospital, at 140, Strand, with the words, "Students' Column," written in the left hand top corner of the envelope.]

### THE STUDENTS' BOOKSHELF.

### "Aphorisms in Applied Anatomy and Operative Surgery."*

This work now before us professes to be merely a book of aphorisms, and, taken as such, we have little but praise for it. A book of this kind may, however, become a source of danger to some students, who may be tempted to rely entirely upon it for their examinations, although carefully warned against doing so in the preface. On the other hand, to the student who has learnt his anatomy thoroughly from some general text-book, who has verified the statements made on the living body or the dead subject, and has gone through a careful course of operative surgery, it presents the ideal synopsis for examination purposes. The author states, "What has been aimed at is a *suggestive* sketch or outline," and he has, we think, succeeded in his object. He further adds, "The aphorisms are not presented as a superstructure, but as a foundation to be builded upon." At the end of the preface he points out those parts of ordinary descriptive anatomy to which the student preparing for the final examination should pay special attention, and enumerates those parts of his popular "Anatomy Tablets" to which they correspond; but, although he does not sufficiently emphasize the point, he at least implies that no part of ordinary general anatomy should be neglected. The work itself is divided into three parts: (1) Applied anatomy; (2) operative surgery; and (3) *vivâ voce* questions in surface-marking, &c. The first part includes, in a crude form, most of the leading points of surgical anatomy, but, as the author himself informs us in the preface, there are "many noticeable omissions," his test as to whether a given statement should or not find place in the "Aphorisms," having been the question "whether the intelligent and diligent student, reading it in his text-book, would *underline it*, or, hearing it, would *jot it down in his note-book*." We certainly consider that the intelligent student would make some notes on the important subject of the anatomy of fractures and dislocations, but Mr. Cooke doubtless thinks that this matter is sufficiently dwelt on in surgical text-books. We further notice that in a few points there is a slight departure from the writings of other authors; but this experienced teacher must be accepted as an authority on the subject. This part concludes with a reprint of those parts of the "Anatomy Tablets" which deal with hernia and the perineum, which will be found very useful. The second portion commences with some general remarks on ligatures and amputations, which are exceedingly good, being brief and to the point, and form one of the best features of the book. With regard to the operations themselves, the author, in his preface, says: "All who have worked at operative surgery will have noticed how circuitous and laboured must necessarily be the written description of many procedures, *e.g.*, the proper way of holding a knife, which, on the contrary, are of quite easy demonstration to the assembled class. The aphoristic method *excludes all such matter*, with a view to its being transferred to the tutorial class." The author is doubtless right here, but, on the other hand, should the student be asked to describe a certain operation on paper, a much fuller description would be required than is found in this book, which, from his general reading, he should have no difficulty in doing. However, as a synopsis, the accounts given will be found very useful, but in the case of the operations on the female organs the aphoristic method is carried to such an extent as to render this small portion, to say the least of it, "brief." The third and last part consists of a 100 typical *viva voce* questions in surface-marking, &c., and will be found as useful as the other portions of the book, if not more so. Mr. Cooke having for so many years devoted the whole of his time to teaching and preparing students for examinations, especially of the London Colleges, is now in a position to know what is, and what is not useful for this purpose. Perhaps no one has been so successful in guiding students through the mysteries connected with examinations in general, and London ones in particular. Many men who have repeatedly failed when going up from their medical schools have been successful when they have been under the eye of this well-known teacher. In conclusion, we can most highly recommend the work to every

* "Aphorisms in Applied Anatomy and Operative Surgery." By Thomas Cooke, B.A., B.Sc., M.D. (Paris), F.R.C.S. (Eng.). (Longmans, Green, and Co., London and New York.)

### The Hospital Advertiser.

THE STUDENT OF MEDICINE—(*continued*).

student preparing for his final examination, and expect as great a success for this well-deserving little volume as the author's well-known "Anatomy Tables" has attained.

### APPOINTMENTS.

At the Birmingham General Hospital, Robert M. Simon, M.D. Cantab, M.R.C.P. Lond., M.R.C.S., has been appointed honorary physician. At Bethlem Hospital, James MacDonald Gill, M.B. Lond., has been appointed resident clinical assistant. At Bridgwater Infirmary, Thomas D. H. Holmes, M.B., C.M. Edin., has been appointed house surgeon. At Belfast Royal Hospital, John Smyth Morrow, B.A., M.B., has been appointed house physician. At the Clapham Maternity Hospital, Minnie Madgshon, M.B. Lond., has been appointed resident house surgeon; and Mary Smith, L.R.C.P. and S. Edin., L.F.P.S. Glasgow, has been appointed assistant physician. At the Edinburgh Royal Maternity and Simpson Memorial Hospital, T. S. Balfour, M.B., C.M. Edin., has been appointed house surgeon; H. G. Langwill, M.B., C.M. Edin., has been appointed house surgeon; R. M. Murray, M.B., C.M. Edin., F.R.C.P. Edin., F.R.S.E., has been appointed assistant physician; and C. E. Underhill, M.B. Cantab., F.R.C.P. Edin., M.R.C.S., has been appointed physician. At the Deaconesses Institution and Hospital, Tottenham, Olaf Kloster, M.R.C.S., L.R.C.P., has been appointed resident house surgeon. At the East Suffolk and Ipswich Hospital, Herbert Fox, M.B., B.C. Camb., has been appointed house surgeon. At Hanwell Asylum, Robert L. S. Nuthall, M.R.C.S. Eng., L.R.C.P. Lond., has been appointed fifth assistant medical officer. At the Crumpsall Infirmary, Manchester Workhouse, W. J. Potts, M.R.C.S., L.R.C.P., has been appointed assistant medical officer, and resident medical officer to the New Bridge Street Wards. At the South Devon and East Cornwall Hospital, A. W. F. Sayres, M.R.C.S., L.R.C.P., has been appointed assistant house surgeon. At the General Hospital, Nottingham, Joseph Thompson, L.R.C.P., M.R.C.S., has been appointed surgeon. At the Miller Hospital, Ernest Clarke, M.D., B.S., has been appointed surgeon to the eye department. At the Hope Infirmary, Salford, W. A. M. Garry, L.K.Q.C.P., L.R.C.S. Ireland, has been appointed assistant medical officer. At the Pembrokeshire and Haverfordwest Infirmary, Percival A. Lloyd, F.R.C.S. Eng., has been appointed surgeon. At Anderson's College Medical School, Glasgow, John McIntyre, M.B., C.M. Glasgow, has been appointed lecturer on diseases of the throat and nose. F. W. Burton-Fanning, M.B., Cantab (formerly known as F. W. Burton), late House Physician at Addenbrooke's Hospital, Cambridge, appointed Honorary Physician to Norfolk and Norwich Hospital, Norwich.

### VACANCIES.

The following vacancies are announced this week, the amount of salary in each case being given after the name of the institution:

**Resident Medical Officers.**

Cancer Hospital, Fulham Road, S.W., House Surgeon and Assistant House Surgeon and Registrar—Salaries, £60 and £50, &c.
City of London Lunatic Asylum, Stone, near Dartford, Kent, Clinical Assistant—Board, Lodging, &c.
Great Yarmouth Hospital, House Surgeon—Salary, £90, &c.
Nottingham Borough Asylum, Mapperley Hill, Clinical Assisstant.
Sussex County Hospital, Brighton, House Physician and Assistant House Surgeon.
Sick Children's Hospital, Newcastle-on-Tyne, Resident Medical Officer—Salary, £60, &c.
West Herts Infirmary, Hemel Hempstead, House Surgeon and Dispenser, who shall be also Assistant Secretary—Salary, £100, &c.
York Dispensary, Resident Medical Officer—Salary £130, &c.

**Non-Resident Medical Officers.**

Birmingham and Midland Free Hospital for Sick Children, Steelhouse Lane, Birmingham. Physician—Salary, £60.
Coventry Provident Dispensary, Surgeon.
Hospital for Women, Soho Square, Clinical Assistants.
Hulme Dispensary, Manchester, Surgeon.
Jenny Lind Infirmary for Sick Children, Norwich, Physician.
King's College Hospital, Sambrooke Medical Registrar, Demonstrator of Public Health.
New Hospital for Women, 144, Euston Road, a Qualified Medical Woman as Assistant Physician; two Clinical Assistants.
Parish of Kennoway, Fifeshire, Medical Officer.
Queen's College, Birmingham, Professor of Medicine.

### SCHOLARSHIPS AND PRIZES.

**University of Aberdeen.**

LIST OF STUDENTS WHO GAINED PRIZES, MEDALS, AND CERTIFICATES IN THE FACULTY OF MEDICINE.

ANATOMY.—FIRST YEAR STUDENTS—WINTER BEGINNERS—OSTEOLOGY.

*First Class Certificates of Merit.*—Alexander Don, M.A., Strathdon (prize), 93 per cent.; William Thomson, Cullen, 89·5; Alexander L.

### The Hospital Advertiser.

### THE STUDENT OF MEDICINE—(*continued*).

Cobban, M.A., Aberdeen, 88·5; James Dawson, M.A., Lonmay, 85; Thomas M'K. Ross, Aberdeen, 84·5; George A. Gibb, Cruden, 77·5; Joseph H. Rowe, Cornwall, 76; James A. Black, M.A., Aberdeen, 75.

*Second Class Certificates of Merit.*—William S. E. Waring, Devonshire, 71; Alexander Reid, Portgordon, 69·5; John A. Gordon, M.A., Dingwall, 68·5; John S. Purdy, Morpeth, 68; William Cartwright, Blackburn, 67·5, George Reid, Buckie, 63·5; James M. Keith, Montrose, 62; Patrick Grant, M.A., Grantown, 61·5; James A. Mearns, Kinellar, 60·5.

### First Year Students.

*First Class Certificates of Merit.*—Alexander Don, M.A., Strathdon (prize), 94·5 per cent.; Arthur H. Lister, B.A., Essex (prize), 92·5; John M. H. MacLeod, M.A., Dundee (prize), 91; William Hossack, Banff, 89.5; John S. Marr, Drumoak, 86·5; William Thomson, Cullen, 83·5; George A. Troup, Rhynie, 83; Robert Cruden, M.A., Fetternear, Inverurie, 82·5; George C. Grant, Dufftown, 82; William E. G. Duthie, M.A., Woodside, 80; Lockhart Adam, Banchory, 79 5; Thomas J. Bonner, M.A., Aberdeen, and James S. Laing, Aberdeen—equal, 79; James Dawson, M.A., Lonmay, George Grant, Grantown, and George Thomson, Montrose—equal, 78 5; Andrew D. Kelly, Aberdeen, 78; George A. Gibb, Cruden, Alexander Low, Old Machar, and Archibald Ramsay, M.A., Stonehaven—equal, 76·5; William S. E. Waring, Devonshire, and Alexander Wood, M.A., Stonehaven—equal, 76; James A. Black, M.A., Aberdeen, 75.

*Second Class Certificates of Merit.*—Alexander Archibald, M.A., Buckie, 74; Alexander L. Cobban, M.A., Aberdeen, and Alexander P. Rust, Peterculter—equal, 72·5; James L. Salmond, M.A., Aberdeen, 72; John G. Jones, Elgin, Edward M. Payne, Aberdeen, and David Ross, Aberdeen—equal, 71·5; William Cartwright, Blackburn, 69·5; Robert Smith, Friockheim, 69; John A. Gordon, M.A., Dingwall, 68 5; William Kinlock, Garmouth, 68; George Reid, Buckie, 67·5; William M. Keith, Turriff, 65·5; James A. Mearns, Kinellar, 64; Ernest Barnes, Manchester, 63; Joseph H. Rowe, Cornwall, 62; Alexander Reid, Portgordon, 60·5; Patrick Grant, M.A., Grantown, 60.

### Second Year Students.

*First Class Certificates of Merit.*—Ashley W. Mackintosh, M.A., Aberdeen (prize), 90·5 per cent.; Alfred A. Moore, Havant, Hants (prize), 87; John Ingram, Kennethmont, 85·5; John R. Kennedy, Tain, 84·5, Alexander Brown, M.A., B.Sc., Ordiquhill, 82·5; John Matheson, M.A. Plockton, Stromeferry, 82; William Hector, Aberdeen, 80·5; Alexander Duncan, Glass, and Frank A. Gill, Wick—equal, 78; Adam L. Cruickshank, Fraserburgh, 77; Robert Burgess, Aberdeen, 76·5.

*Second Class Certificates of Merit.*—Charles W. Profeit, Balmoral, 74; Claud K. Morgan, Arbroath, 73·5; James Aldred Allwood, Jamaica, Hugh Fraser, Aberdeen, and Albert Henderson, Aberdeen—equal, 72·5; Frederick S. Ainley, Huddersfield, 72; C. J. Milne, Aberdeen, 70; Robert Mitchell, Fyvie, and George Sturrock, Aberdeen—equal, 69·5; Charles Kemp, Aberdeen, 69; Louis O. Crosswell, Jamaica, 68; Charles L. Morrison, Rothes, 67·5; Donald G. Falconer, Cullen, 67; P. M. Muttukumaru, Ceylon, 65·5; G. G. M. Black, Cruden, George Marr, Oldmeldrum, and Alexander Ogston, equal, 64·5; Edward Philip, Aberdeen, 63; Alexander Thomson, Fochabers, 61·5; William C. Edmonston, Aberdeen, James F. Philip, Skene, and A. Ponnambulam, Ceylon, equal, 61; Hector Munro, Fortrose, Charles E. Maguire, Aberdeen, and Richard A. Slater, Preston, equal, 60.

### Advanced Students.

*First Class Certificates of Merit.*—John T. West, Dinnet (prize), 93 per cent.; John W. Grant, Watten, Caithness (prize), 89; Wm. R. Pirie, M.A., Aberdeen, 79·5; William M. Anderson, Alford, 79; Andrew Davidson, Forres, 78; Alfred T. Smith, Banff, 77·5; James Rust, M.A., Aberdeen, 76 5.

*Second Class Certificates of Merit.*—Andrew Baxter, Aberdeen, 74; William Moir, Forgue, 73·5; Alexander Grant, Dufftown, 73; Charles G. Macgregor, Banff, 72·5; Harry M. Mitchell, Tyrone, 72; James Wallace, Fearn, Ross-shire, 71·5; Thomas Brander, Garmouth; Robert R. Sutter, Timaru, New Zealand, and William John Carmichael, Aberdeen—equal, 71; William Ross, Fearn, Ross-shire, and William Sinclair, New Pitsligo—equal, 68; Thomas H. Galbraith, London, 67; John Leach, Arderseir, and Claud W. Marshall, Greenock—equal, 66; Alexander Fraser, Inverness, 65; Duncan D. Mackintosh, Aberdeen, 64·5; Alfred Hogg, Laurencekirk, and William A. Justice, Aberdeen—equal, 64; Leslie Valentine, Inverurie, 63·5; Andrew W. Thomson, Jamaica, 63; Alexander Anderson, Elgin, 62·5; Frank Beaton, Inverurie, and William A. G. Russell, Longhope, Orkney—equal, 61·5; William J. S. Ewan, Fyvie, 61; Robert E. Kerr, Bunchrew, Inverness, 60·5; John Cameron, Beauly, 60.

### CHEMISTRY.—First Year Students.

*Silver Medallist.*—Robert Cruden, M.A., Inverurie, 95 per cent.

*Bronze Medallist.*—Arthur H. Lister, B.A., Leytonstone, Essex, 91.

*First Class Certificates.*—David Ross, Aberdeen, 83; William Cockburn, New Deer, 80.

*Second Class Certificates.*—William E. G. Duthie, M.A., Woodside, and Robert Smith, Friockheim, 71; Alegander Archibald, M.A., Keith, 69; Joseph H. Rowe, Hayle, Cornwall, 64; Alexander P. Rust, Peterculter, 63; Andrew T. Gage, Aberdeen, and R. M. Litt,ejohn, New Deer, 62; Ernest Barnes, Manchester, Andrew Kelly, Aberdeen; William Philip, M.A., Inverurie, and George Alexander Troup, Rhynie—equal 60.

### Second Year Students.

*Bronze Medallist.*—Alexander Don, M.A., Strathdon, 83 per cent:

*First-Class Certificates.*—William Thomson, Cullen, 80 per cent.; Peter Mackessack, Forres, 79; George Bruce, M.A., Inverurie, 77; William Thomson, Turriff, 76.

*Second-Class Certificates.*—William M. Keith, Turriff, 64 per cent.; John H. Goodliffe, Nottingham, 61.

### THE STUDENT OF MEDICINE—(*continued*).

INSTITUTES OF MEDICINE.—Senior Division.

*Medallists.*—John W. Grant, Watten, Caithness, 83 per cent.; Duncan D. Mackintosh, Aberdeen, 81; Robert E. Kerr, M.A., Bunchrew, Inverness, and William R. Pirie, M.A., Aberdeen, 80.

*First Class Certificates.*—William J. S. Ewan, Fyvie, and Robert Ferguson, M.A., Aberdeen—equal, 78; William R. Duguid, M.A., Buckie, 77; William Boddie, M.A., Aberdeen, 76; William M. Anderson, Alford, Thomas Brander, Garmouth, Alfred T. Smith, Banff, and William Trethowan, Victoria, Australia—equal, 75.

*Second Class Certificates.*—John T. West, Dinnet, 72; Alexander Grant, Dufftown, 71; James Rust, M.A., Aberdeen, 69; Robert H. Marshall, Aberdeen, 65; Carles M. Dawson, Rathen, Lonmay, and William Ross, Fearn, Ross-shire—equal, 63; Alexander Fraser, Inverness, 62; Claud W. Marshall, Greenock, 61; William J. Carmichael, Aberdeen, William Moir, Hawkhall, Forgue, William Sinclair, M.A., New Pitsligo, and James Sutherland, Newcastle-on-Tyne—equal, 60.

Junior Division.

*Medallists.*—Ashley, W. Macintosh, M.A., Deskford, Cullen, and William A. G. Russell, M.A., Longhope, Orkney—equal 88 per cent.; A. M. R. Sinclair, Inverness, 86; Robert R. Sutter, New Zealand, 83.

*First Class Certificates.*—James Wallace, Fearn, Ross-shire, 78; Andrew W. Thomson, Jamaica, and George A. Williamson, M.A., Inverness—equal, 75.

*Second Class Certificates.*—Edward Griffiths, Clocaenog, Wales, and Alex. Stables, Old Aberdeen—equal 69; Andrew Davidson, Forres, and Duncan Finlayson, Tain—equal 67; James Ritchie, Fintray, 63; A. Rajasingham Ceylon, William H. de Silva, Moratua, Ceylon, and Thomas W. Youngson, Aberdeen—equal 60.

SYSTEMATIC SURGERY.—Senior Division.

*Prize Medal and First Class Certificate.*—James Rust, M.A., 91·5 per cent.

*Medal and First Class Certificate.*—John Leach, M.A., 88·4; Alfred T. Smith, 86; James Sutherland, 85·4; Alfred Hogg, M.A., and William A. Pirie, M.A.—equal, 83·1; William Trethowan, 82·2; William Sinclair, M.A., 81·5.

*First Class Certificate.*—John T. West, 78·7; William A. G. Russell, M.A., 76; William R. Duguid, M.A., 75·3.

*Second Class Certificates.*—John W. Grant, 73·9; Thomas Brander and Alexander Fraser—equal, 73·7; Charles M. Dawson, 71 7; Frank B. Graham, 71·4; Thomas H. Galbraith, 70·8; William Grant, 70·2; Leslie Valentine, 69·4; Claude W. Marshall, 68·6; Arthur W. Paterson, 67·7; Robert R. Sutter, 67·1; Alexander G. Gall, 67; William M. Anderson, 66·4; Henry T. Dawson, 66 2; Frank Beaton and Robert E. Kerr, M.A.—equal, 66; William Ross, 64·5; Andrew Davidson, 64 3; George Geddes, 63·2; James Wallace, M.A., 62·4; William Moir, 62·1; William J. S. Ewan, 62; James Ritchie, 61·4; Arthur B. Dalgetty, 60·7; George Savege, 60·2; John G. Pardoe, 60; Albert Brown, 60.

Junior Division.

*Medal and First Class Certificates.*—Robert Mitchell, 89·8; Peter Howie, 88·9.

*First Class Certificates.*—Adam Alexander, 82·6; Frank A. Gill, 79·9; Alexander Brown, M.A., B.Sc., 79·1.

### PASS LISTS.

### Examining Board in England by the Royal Colleges of Physicians and Surgeons.

The following gentlemen passed the second examination of the Board at a meeting of the examiners on the 16th inst.:—

Anatomy and Physiology.—E. F. Herman Hardenberg. H. Allcroft Duffett, and John A. Howard, of Guy's Hospital; Aslett Baldwin, of Middlesex Hospital: T. Harold Woodfield, of St. Bartholomew's Hospital; R. Llewelyn Jones and Thomas Harrison, of University College; Edward W. Joscelyne, of St. Mary's Hospital; Arthur F. Goldsmith, of St. George's Hospital; Joseph A. Edwards, G. Moore Hetherington, J. Smedley Boden, and William R. Bryett, of King's College; and G. Mansell Dawkin, of London Hospital.

Anatomy Only.—A. Morris Wilkinson, of Westminster Hospital; A. Godfrey Ince and William Escombe, of Charing Cross Hospital; Courtney C. Parsons, of St. Mary's Hospital; W. Hartland Horton, of St. Bartholomew's Hospital; and R. Ardra Fegan, of St. Bartholomew's Hospital and Mr. Cooke's School of Anatomy and Physiology.

Physiology Only.—Oswald O. Williams and J. Grant Fowler, of London Hospital; John Grech, of St. Mary's Hospital; Henry Hewetson, of Guy's Hospital; and William J. Robertson, of Charing Cross Hospital.

Passed on the 17th inst.:—

Anatomy Only.—H. Wrangel Clarke and Edward S. Ch'lcott, of St. Mary's Hospital; George H. Steele, Augustus White, and Edwin S. Hoare, of Guy's Hospital; C. Milner Spain, W. Sidney Mayne, and Maxwell Dick, of University College; Samuel R. Wright, Mervyn T. Archdall, Ernest Parsons, and Percy C. Spark, of Charing Cross Hospital; R. Wentworth Dillon, C. Westbrooke Grant-Wilson, Henry W. Oborn, and F. W. Jervis Goodhue, of St. Thomas's Hospital; Charles Corben, of St. Bartholomew's Hospital; and G. Read Hannon, of King's College Hospital.

In Anatomy and Physiology 320 candidates presented themselves, of whom 184 passed in both subjects; 45 in Anatomy only, and 35 in Physiology only; 56 were referred in both subjects. In Anatomy only, 29 candidates presented themselves, 21 of whom passed; in Physiology only, there were 28 candidates, of whom 24 passed.

